# The Effect of Long-Term Continuous Cropping of Black Pepper on Soil Bacterial Communities as Determined by 454 Pyrosequencing

**DOI:** 10.1371/journal.pone.0136946

**Published:** 2015-08-28

**Authors:** Wu Xiong, Zhigang Li, Hongjun Liu, Chao Xue, Ruifu Zhang, Huasong Wu, Rong Li, Qirong Shen

**Affiliations:** 1 Spice and Beverage Research Institute, Chinese Academy of Tropical Agricultural Science, Wanning, Hainan 571533, China; 2 National Engineering Research Center for Organic-based Fertilizers, Jiangsu Key Lab for Solid Organic Waste Utilization, Jiangsu Collaborative Innovation Center for Solid Organic Waste Resource Utilization, Nanjing Agricultural University, 210095, Nanjing, China; 3 Key Laboratory of Microbial Resources Collection and Preservation, Ministry of Agriculture, Institute of Agricultural Resources and Regional Planning, Chinese Academy of Agricultural Sciences, Beijing 100081, P. R. China; Chinese Academy of Sciences, CHINA

## Abstract

In the present study, 3 replanted black pepper orchards with continuously cropping histories for 10, 21, and 55 years in tropical China, were selected for investigating the effect of monoculture on soil physiochemical properties, enzyme activities, bacterial abundance, and bacterial community structures. Results showed long-term continuous cropping led to a significant decline in soil pH, organic matter contents, enzymatic activities, and resulted in a decrease in soil bacterial abundance. 454 pyrosequencing analysis of 16S rRNA genes revealed that the *Acidobacteria* and *Proteobacteria* were the main phyla in the replanted black pepper orchard soils, comprising up to 73.82% of the total sequences; the relative abundances of *Bacteroidetes* and *Firmicutes* phyla decreased with long-term continuous cropping; and at genus level, the *Pseudomonas* abundance significantly depleted after 21 years continuous cropping. In addition, bacterial diversity significantly decreased after 55 years black pepper continuous cropping; obvious variations for community structures across the 3 time-scale replanted black pepper orchards were observed, suggesting monoculture duration was the major determinant for bacterial community structure. Overall, continuous cropping during black pepper cultivation led to a significant decline in soil pH, organic matter contents, enzymatic activities, resulted a decrease in soil bacterial abundance, and altered soil microbial community membership and structure, which in turn resulted in black pepper poor growth in the continuous cropping system.

## Introduction

Soil microbial communities are responsible to soil functions and the ecosystem sustainability [1,2]. Many previous studies have shown that soil microbial community membership and structure are key determinants on soil health and can be affected by various agricultural management factors, including crop rotation and tillage [3], fertilizer regime [4], pesticide application [5], irrigation [6], and continuous cropping [7].

Continuous cropping refers to a system in which certain crops are ‘‘replanted” in soils that had previously supported the same or similar plant species [8]. Because of limited arable lands and expansive populations in China, continuous cropping systems are commonly practiced in the production of grain crops [9,10] and cash crops [11,12]. However, long-term continuous cropping usually leads to plant growth inhibition and serious soil-borne diseases [13,14], which has been described as a continuous cropping obstacle (also known as replanting disease).

Black pepper (*Piper nigrum* L.) is one of the most widely used spices with high economic value in tropical agricultural regions [15]. Hainan island, a typical tropical area where black pepper can grow well, produces 90% of the black pepper in China [16]. However, long-term continuous cropping causes poor growth, low yield, and serious soil-borne disease [16], thus severely hindering the black pepper industry in China.

Various factors have been considered to induce continuous cropping obstacles, including deterioration of soil physicochemical properties, decrease of soil enzymatic activities, accumulation of autotoxic substances, and build-up of the soil-borne pathogens [17,18]. Recently, increasing numbers of studies have speculated that the disruption of the soil microbial community also contributes to the continuous cropping obstacles after long-term continuous cropping [19,20]. However, the detailed effects of continuous cropping on soil microbial communities and the link between these effects and soil productivity remain unclear [21]. In addition, to our knowledge, very few studies documented on the variations of soil microbial communities in black pepper continuous cropping system [22] and their effects on soil microflora and the black pepper growth.

Bacteria, the most diverse and abundant soil organisms [23] and are good indicators for plant heath for many bacterial groups have been identified as bio-control agents against soil-borne pathogens and play key roles in plant growth promoting [24,25]. Previous studies regarding the effects of continuous cropping systems on soil bacteria communities were based on 16S rRNA gene library construction or fingerprinting methods [26,27]. However, these strategies are laborious, costly, time consuming, and only detect certain dominant soil microbial groups. Recently, the next-generation sequencing (NGS) technologies, such as 454 deep amplicon sequencing [28] provides an unprecedented opportunity for achieving a high throughput and deeper insight into soil bacterial communities.

In this study, 3 replanted black pepper orchards with continuously cropping histories for 10, 21, and 55 years in tropical China, were selected to investigate the effect of long-term continuous cropping on soil physiochemical properties, enzyme activities, bacterial abundance, and bacterial community structures. The objective of the our study was 1) to explore potential correlation between soil physiochemical properties, enzyme activities, and soil productivity, 2) to provide a better understanding of soil bacterial communities on black pepper growth under continuous cropping system.

## Materials and Methods

### Sampling sites

The experimental site is located at the Spice and Beverage Research Institute, Wanning City, Hainan Province, China (110°19'E-110°22'E, 18°72'N-18°76'N). The elevation, annual rainfall, and mean annual temperature are 26 m, 2201 mm, and 24.5°C, respectively. The experimental soil samples were collected on March 15, 2013 from 3 black pepper orchards containing the same black pepper cultivar (*Piper nigrum* L. cv. Reyin No. 1) that were continuously for 10, 21, and 55 years, referred as “10y”, “21y”, and “55y” respectively below. The 3 black pepper orchards ranged from about 1900 m^2^ to 2100 m^2^ in size. It is worth noting that from our previous field investigation, obvious poor growth and low yield of black pepper were shown over 10 years continuous cropping (S1 Table). The agronomic management and fertilization regime were similar in the 3 black pepper orchard sites. For each black pepper orchard, three biological samples were randomly selected and each biological sample was a composite of 15 soil cores randomly collected at 60 ± 10 cm from black pepper trunk (2.5 cm in diameter, 0–30 cm in depth). All 9 soil samples (3 fields × 3 replicates) were put into sterile plastic bags, placed into ice box, and transported to the laboratory. After passing through a 2 mm sieve, each sample was divided into two subsamples: one portion was air-dried for soil characteristic and soil enzyme activity analysis; the remainder was stored in a refrigerator at -80°C for the DNA extraction.

### Pot Trials

Pot experiments were performed to evaluate the effects of the soils from 3 black pepper orchards on the seedling black pepper growth. Uniform black pepper seedling clones collected from the rapid multiplication nursery with four nodes were used. Each pot contained 4.5 kg of soil and one black pepper seedling clone. Each treatment had triplicates (three blocks), and each block had 9 pots. All the pots were randomly placed in the greenhouse with average temperature of 30°C and average humidity of 72%. After 4 months, root length, shoot weight, shoot height, and root activity of the black pepper plants were measured. The root activity was measured according to the triphenyl tetrazolium chloride (TTC) method [29], which was showed as TTC reduction intensity, *i*.*e*., the amount of TTC reduction (μg)/fresh root weight (g) × time (h).

### Soil physiochemical properties and soil enzyme activities determinations

The soil physiochemical properties were determined according to our previous method [30]. In short, soil pH was determined using a glass electrode meter in soil suspension with deionized-distilled water (1:5 w/v). Organic matter (OM) was measured according to the potassium dichromate external heating method and available N (hydrolyzable N) were measured with the alkaline-hydrolyzable diffusion method. Soil available P was extracted with sodium bicarbonate and determined by the molybdenum blue method. Soil available K was extracted with ammonium acetate and then measured using flame photometry.

The activity of soil urease, sucrase, and catalase was detected according to Alef and Nannipieri [31] with some modifications. Briefly, the urease activity in soil was determined though incubating 5.0 g soil in the 10% urea solution (10 ml) at 37°C for 24 h. Soil sucrase activity was assessed with 5.0 g soil incubating in the sucrose solution at 37°C for 24 h. Soil catalase was analyzed by titration with KMnO_4_ method [32].

### DNA extraction, 16S rRNA gene amplification, and pyrosequencing

For each soil sample, DNA extraction from 500 mg soil was carried out with a MoBio PowerSoil DNA Isolation Kit (Mo Bio Laboratories Inc., Carlsbad, CA, USA) following the manufacturer’s protocol. Total DNA quality and concentration were measured by the NanoDrop ND-2000 spectrophotometry (NanoDrop Technologies, Thermo Scientific, USA). Primer set of F515 (GTGCCAGCMGCCGCGG) and R907 (CCGTCAATTCMTTTRAGTTT) were chosen to amplify the V4-V5 hypervariable regions of bacterial 16S rRNA gene [33], which covered over 98% of the 16S gene sequences in the ribosomal database. The Roche adapter A and a unique 7-bp barcode were added at the 5' end of the F515 primer and the Roche adapter B was added at the 5' end of the R907 primer for the 454 pyrosequencing. Detailed information regarding barcode sequence is shown in S2 Table. The PCR reactions for each sample were conducted in triplicate following our previous publication [30]. The triplicate products were purified using a Qiagen QIAquick Gel Extraction kit (Qiagen Gmbh, Germany), and then pooled in a equimolar concentration of 10 ng ul^-1^ for pyrosequencing, which was performed on the 454 GS-FLX + sequencer (454 Life Sciences) at Personal Biotechnology Co., Ltd (Shanghai, China).

### Quantification of total bacterial abundance in soil

Real-time quantitative polymerase chain reaction (qPCR) was performed according to Chen et al. [34] for quantification the soil bacterial abundance. Three replicates of each sample was used to determine the copy number of the bacterial 16S rRNA gene with the primers F515 and R907 using the ABI 7500 Cycler (Applied Biosystems, Germany). The PCR amplifications were carried out in a total volume of 20 μl, containing 10 μl of the *Premix Ex Taq* ^*TM*^ (2×) (Takara), 0.4 μl of ROX Reference Dye II (50×), 0.4 μl of each primer (10 μM), 2 μl of template DNA, and 6.8 μl of ddH_2_O. All amplifications were conducted with the following thermal conditions: an initial denaturation step at 95°C for 30 s; followed by 40 cycles of 95°C for 5 s, 60°C for 34 s, as described by Chen et al. [34]. The standard curve was generated using 10-fold dilution series of plasmid DNA containing a fragment of the 16S rRNA gene from the *Bacillus subtilis* 168.

### Pyrosequencing data analysis

Raw bacterial sequence reads were treated and analyzed using Mothur v. 1.25.1 with the Schloss SOP pipeline [35]. Briefly, low quality sequences with an average quality score less than 25, length shorter than 200 bp, any mismatches to the primers, or a homopolymer longer than 8 bases were excluded. The passing sequences were assigned to the individual sample according to the 7-bp barcodes, and the remaining sequences were clustered into OTUs with a cutoff of 97% similarity. Then, the representative OTU sequences were classified against RDP database with the 60% confidence threshold. Finally, singleton OTUs that contain only one sequence in all 9 samples were removed for the subsequent analyses.

### Statistical analyses

Coverage, richness (Chao1 and ACE indexes), and diversity (Shannon indices) were used to estimate the alpha diversity of each sample. Hierarchical cluster dendrograms (with Bray-Curtis distance dissimilarities) were performed to compare the bacterial community structures across all the soil samples in Mothur [35]. The correlations between the abundant bacterial phyla and soil characteristics were determined by the Mantel test and redundancy analysis (RDA) was carried out via the vegan package of R. For other parameters, one-way analysis of variance (ANOVA) with Turkey’s HSD test were conducted for multiple comparisons, and the Spearman’s rank correlations were calculated using SPSS v20.0 (SPSS Inc., USA).

### Sequence accession numbers

All raw sequences data were accessible in NCBI Sequence Read Archive (SRA) database under the accession number SRA149260.

## Results

### The physicochemical characteristics and enzyme activities in the 3 replanted black pepper orchards soils

The soil pH and organic matter (OM) content deceased with the long-term continuous cropping of black pepper, while the available N and available P contents increased overtime (Table 1). Soil urease, catalase, and sucrase activities were conducted to measure the potential turnover rates of nitrogen or carbon in the 3 replanted black pepper orchards soils. As shown in Table 2, soil sucrase activity deceased with black pepper continuous cropping, and among the 3 replanted black pepper orchards, the “10y” orchards showed the highest soil catalase activity. However, the urease activity did not differ significantly among the 3 replanted black pepper orchards soils.

**Table 1:** Soil characteristics of the black pepper orchards.

Black pepper orchards	pH	OM (g/kg soil)	Available N (mg/kg soil)	Available P (mg/kg soil)	Available K (mg/kg soil)
10y	4.90±0.02 a	19.00±0.90 a	130.81±3.05 b	10.04±0.76 c	69.74±5.53 a
21y	4.32±0.13 b	17.35±0.89 a	138.43±4.24 ab	28.40±9.83 b	41.98±3.62 b
55y	4.37±0.12 b	15.17±0.43 b	148.34±5.95 a	63.42±2.29 a	35.95±4.18 b

Values are means ± standard deviation (n = 3). Means followed by the same letter for a given factor are not significantly different (*P* < 0.05; Turkey’s HSD test).

**Table 2:** Soil enzyme activities.

Black pepper orchards	Urease (mg NH_3_-N/g soil/24 h)	Catalase (mL 0.1 mol/L KMnO_4_/g soil/20 min)	Sucrase (mg glucose/g soil/24 h)
10y	0.20±0.01 a	1.08±0.08 a	1.99±0.02 a
21y	0.21±0.02 a	0.34±0.07 c	1.66±0.02 b
55y	0.19±0.01 a	0.58±0.02 b	1.18±0.05 c

Values are means ± standard deviation (n = 3). Means followed by the same letter for a given factor are not significantly different (*P* < 0.05; Turkey’s HSD test).

### Pot experiments

To further analyze the effects of the replanted black pepper orchard soil on black pepper growth, greenhouse trials were performed. The “10y” orchard soil showed the highest shoot dry weight, the longest total root length and the highest root activity of black pepper (Table 3). And the “55y” orchard soil showed the poorest black pepper growth indicators, suggesting that long-term continuous cropping seriously affected the black pepper growth.

**Table 3:** Growth parameters and root activity of seedlings black pepper in pots.

Black pepper orchards	Shoot dry weight (g)	Shoot height (cm)	Total root length (cm)	Root activity (TTC reduction μg root/g/h)
10y	11.31±1.17 a	51.33±0.58 a	430.33±6.50 a	194.28±35.32 a
21y	9.13±0.99 ab	50.33±1.15 a	415.30±7.49 ab	140.75±13.39 ab
55y	8.11±0.65 b	49.00±1.00 a	399.78±6.92 b	88.53±19.72 b

Values are means ± standard deviation (n = 3). Means followed by the same letter for a given factor are not significantly different (*P* < 0.05; Turkey’s HSD test).

### Bacterial abundance

The qPCR results revealed that there were about 5.76×10^9^ to 6.50×10^9^ 16S rRNA genes copies per g of soil among the 3 replanted black pepper orchards (Table 4). The “10y” orchard showed the highest 16S rRNA gene copy numbers (6.50×10^9^ copies g^-1^ soil). In addition, we found that the soil bacterial abundance decreased with the long-term continuous cropping of black pepper.

**Table 4:** Real-time PCR quantification of bacterial 16S rRNA genes.

Black pepperorchards	Bacterial 16S rRNA genes (10^9^ copies/g soil)
10y	6.50±0.31 a
21y	6.05±0.28 ab
55y	5.76±0.17 b

Values are means ± standard deviation (n = 3). Means followed by the same letter for a given factor are not significantly different (*P* < 0.05; Turkey’s HSD test).

### Community composition of bacteria

**Fig 1:**
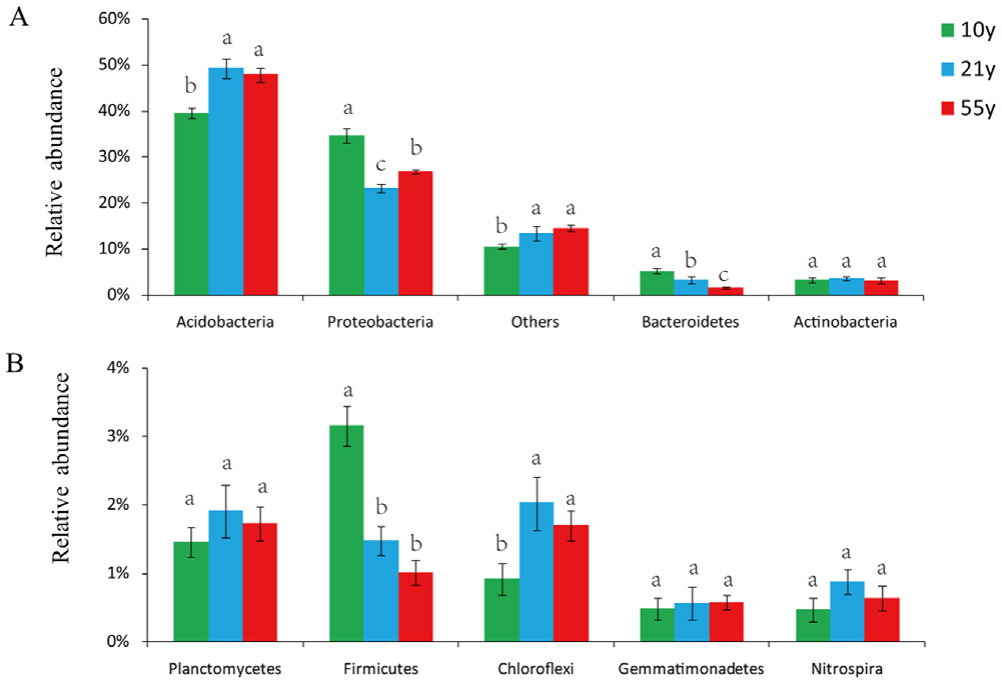
The relative abundances (RA) of bacterial phyla (A: RA > 3%; B: RA 0.1~3%) in the soils of the 3 replanted black pepper orchards. The “others” comprised the unclassified and the low abundance phyla (RA < 0.1%). “10y”, “21y”, and “55y” stand for 3 black pepper orchards with 10, 21, and 55 years’ succession cropping history, respectively. Bars represent the standard error of the three replicates and different letters above each phylum indicate significantly difference at 0.05 probability level according to the Turkey’s HSD test.

**Fig 2:**
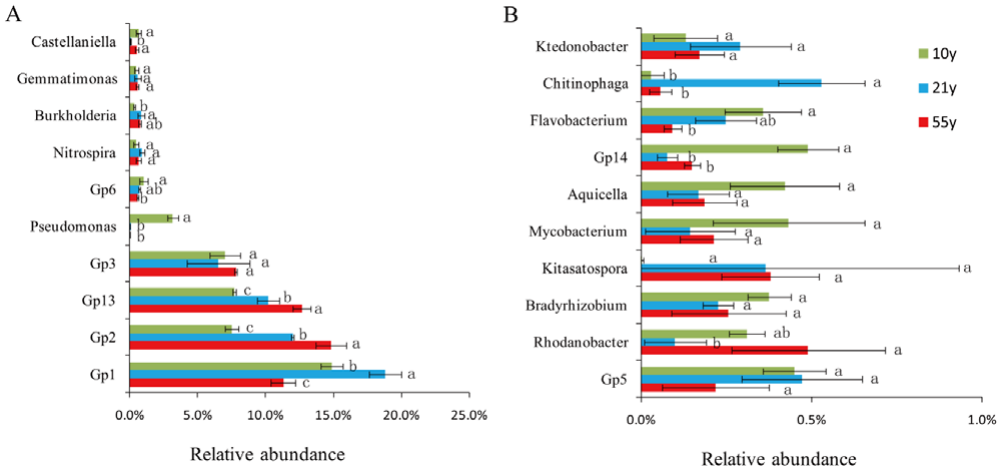
The relative abundances of the top 20 classified bacterial genera (A: top 10 genera; B: top 11–20 genera) in the 3 replanted black pepper orchards. “10y”, “21y”, and “55y” stand for 3 black pepper orchards with 10, 21, and 55 years’ succession cropping history, respectively. Bars represent the standard error of the three replicates and different letters above each genus indicate significantly difference at 0.05 probability level according to the Turkey’s HSD test.

After quality filtering and singleton OTUs removing, 100,122 high quality sequences were gained from the 9 soil samples with the pyrosequencing-based analysis. The classified OTUs from all soil samples were primarily affiliated in the 9 bacterial phyla (Fig 1A and 1B). *Acidobacteria* (45.58% of all sequence reads), *Proteobacteria* (28.23%), *Bacteroidetes* (3.34%), *Actinobacteria* (3.32%), *Planctomycetes* (1.70%), *Firmicutes* (1.88%), and *Chloroflexi* (1.54%) were the dominant bacterial phyla (relative abundance > 1%). Additionally, the relative abundances of *Bacteroidetes* and *Firmicutes* phyla decreased with long-term continuous cropping of black pepper. Compared to the “10y” black pepper orchard soil, “21y” and “55y” orchard had higher relative abundances of *Acidobacteria* and *Chloroflexi*, and lower abundance of *Proteobacteria* (Fig 1A and 1B). Furthermore, comparison of the relative abundances of the top 20 classified bacterial genera showed significant variations among the 3 replanted black pepper orchards soils (Fig 2A and 2B), the relative abundances of *Pseudomonas*, Gp6, and *Flavobacterium* significantly depleted with increasing years of black pepper cropping.

### Bacterial alpha-diversity

Coverage from all samples was above 96%, indicating that sequencing reads were sufficient for this analysis (Table 5). Even though not significantly, the Chao1 and ACE indexes decreased with black pepper consecutive cropping. In addition, Shannon index significantly decreased after monoculture of black pepper for 55 years.

**Table 5:** Bacterial α-diversity indices.

Black pepper orchards	Coverage (%)	Chao1	ACE	Shannon
10y	96.51±0.17 a	1165.26±41.12 a	1154.44±43.67 a	5.35±0.03 a
21y	96.63±0.14 a	1117.08±52.48 a	1129.16±29.63 a	5.46±0.06 a
55y	96.65±0.09 a	1103.32±8.12 a	1093.10±13.99 a	5.17±0.03 b

Values are means ± standard deviation (n = 3). Means followed by the same letter for a given factor are not significantly different (*P* < 0.05; Turkey’s HSD test).

### Bacterial community structure

**Fig 3:**
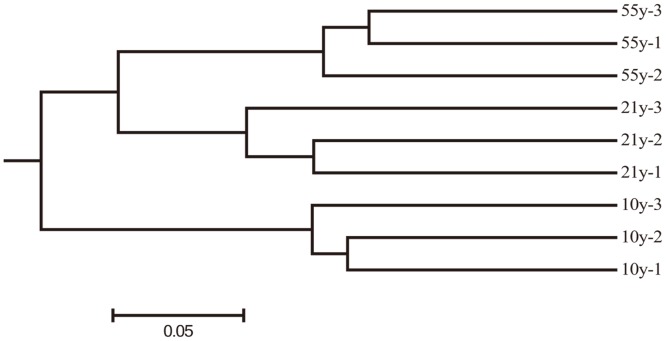
Bray-Curtis dissimilarity hierarchical cluster tree of soil bacterial communities from the 3 replanted black pepper orchards. “10y”, “21y”, and “55y” stand for 3 black pepper orchards with 10, 21, and 55 years’ succession cropping history, respectively.

Bray-Curtis dissimilarity hierarchical clustering analysis showed that soil bacterial community structure in the soil samples collected from the same time-series orchard were more similar as the 3 highly supported clusters were grouped together (Fig 3). Among the 3 black pepper orchards, the “21y” and “55y” orchards grouped together and were separated from “10y” orchard, which suggested the soil bacterial community structure might be affected by the monoculture duration.

### Effects of soil physicochemical properties on abundant phyla

**Fig 4:**
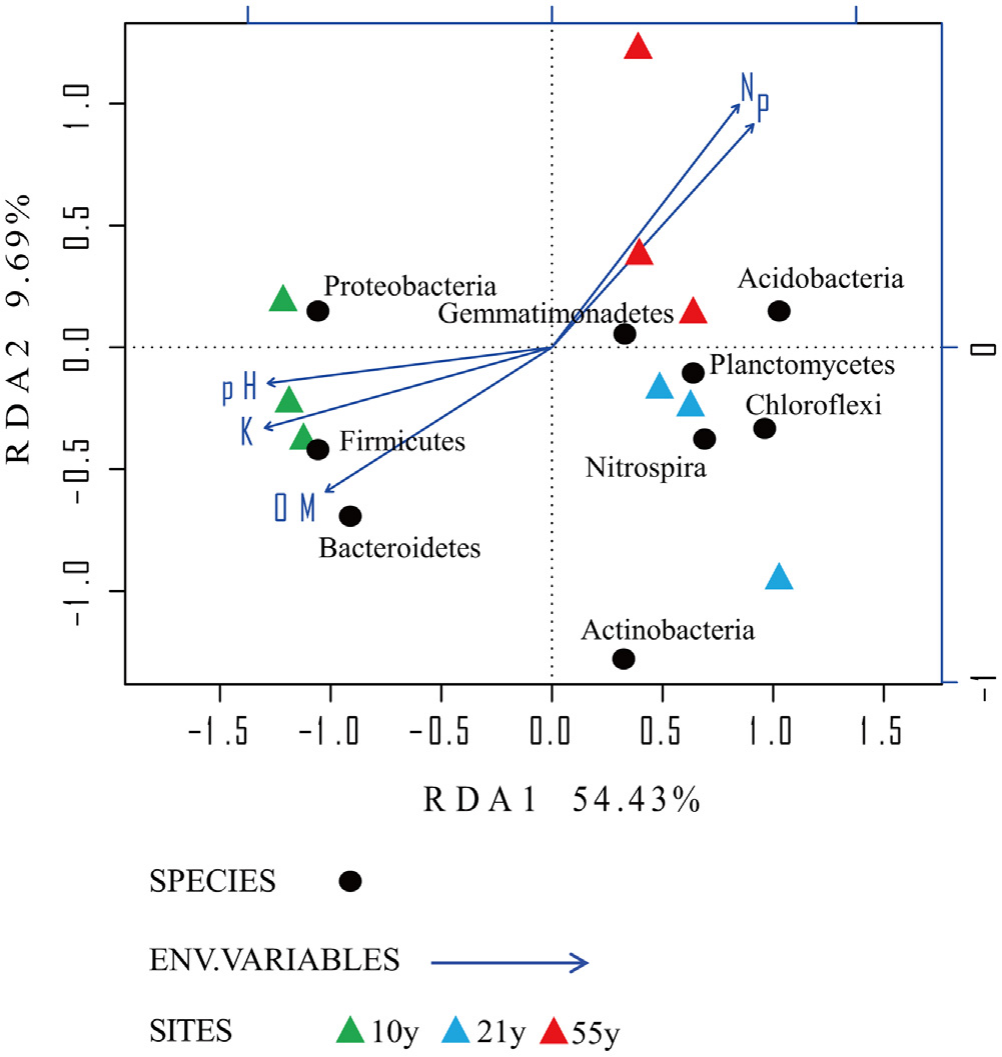
Redundancy analysis (RDA) of the abundant bacterial phyla and environmental variables for individual samples from the 3 replanted black pepper orchards. “10y”, “21y”, and “55y” stand for 3 black pepper orchards with 10, 21, and 55 years’ succession cropping history, respectively.

The Mantel test analysis revealed a significant (*r* = 0.61, *P* < 0.05) correlation between the soil properties and the abundant bacterial phyla. The first two components of RDA explained 54.43% and 9.69% of the total bacterial phyla data variations (Fig 4). The first component (RDA1) separated “10y” orchard samples from the “21y” and “55y” orchards samples, and “21y” orchard was separated from “55y” orchard by the second component (RDA 2).

Moreover, Spearman’s correlation coefficient was used to assess relationships between the soil properties and abundant bacterial phyla (S3 Table). The *Proteobacteria* abundance showed positive relationship with soil pH. The *Bacteroidetes* and *Firmicutes* phyla relative abundances were positively correlated with soil OM and available K, while negatively correlated with soil available N and P.

## Discussion

Knowing the characteristics of soil physicochemical properties and enzyme activities in black pepper continuous cropping systems could be helpful to provide a better understanding of soil productivity in replanted black pepper orchards. Lower soil pH and lower organic matter content were observed in long-term monoculture black pepper system. Long-term application of nitrogen fertilizer [36] and accumulation of allelochemicals [37] might be the key factors leading to soil acidification in black pepper cropping systems. Moreover, in accordance with our results, previous study already reported continuous cropping often results in a decline in soil organic matter [38], this may be attributed to the following factors: reduced organic material returned to the soil and long-term application of chemical fertilizer [39]. With the black pepper cropping years increasing, soil available N and P contents significantly increased, which could be also attributed to long-term oversupply of chemical fertilizer [16]. Soil enzyme activities are considered to be an important indicator of soil quality and ecological stability [40]. In this study, soil catalase and sucrase activities significantly decreased with 55 years of black pepper cropping, the results were consistent with the precious study that continuous monocropping of peanut led to a decrease in soil enzyme activities [41]. The results of present study revealed a significant decline in soil pH, organic matter contents, and enzymatic activities under long-term continuous cropping system, which might limit the black pepper growth in fields.

Replant obstacle properties of black pepper have always been observed at the Tropical Spice and Beverage Research Institute in Hainan island [16]. In our previous field investigation, over 10 years continuous cropping of black pepper, obvious poor growth and low yield were shown. In this study, the results of the pot experiments revealed that the growth of black pepper exhibited significant reductions with the increasing years (Table 3). These results are consistent with many previous studies, such as apple [26] and peach trees [13], the growths of which were significantly hindered in replanted orchard sites. Thus, this observation might be a general phenomenon in continuous cropping agro-ecological systems.

In this study, after quality filtering, 100,122 sequences were obtained from the 3 time-scale replanted black pepper orchards, and the *Acidobacteria* and *Proteobacteria* were the top two abundant phyla (Fig 1A), which generally agreed with many previous articles that *Acidobacteria* and *Proteobacteria* were the most common phyla in different agricultural systems or other soil types though pyrosequencing surveys [42,43]. Significant differences in bacterial community composition in soil samples collected from the 3 time-scale black pepper replanted orchards were observed. The relative abundance of *Acidobacteria* significantly increased in after 21 years black pepper continuous cropping (Fig 1A), which could be attributed to the pH decrease caused by long-term intensive cropping system [44]. The relative abundances of *Bacteroidetes* and *Firmicutes* phyla decreased with long-term continuous cropping of black pepper, which agreed with previous observations [30]. Interestingly, previous study has found that *Bacteroidetes* was very important as an indicator of soil health in vanilla monoculture system [30]. Moreover, the relative abundance of *Firmicutes* corresponded with soil-borne disease suppression [45]. At the genus level, *Pseudomonas* relative abundance significantly depleted with 21 years black pepper continuous cropping (Fig 2A). Previous studies showed that the densities of *Pseudomonas* in the apple rhizosphere, which was closely related to plant growth, being decreased over years after replanting [26,46]. Additionally, *Pseudomonas* was an effective antagonistic endophyte for biological control of *Phytophthora* foot rot in black pepper [47]. These results suggested that continuous cropping of black pepper may significantly decrease the relative abundance of plant-beneficial bacteria, which might be an important factor for black pepper poor growth in the black pepper replanted systems.

The bacterial abundance significantly decreased with increasing years of black pepper cropping. In addition, bacterial diversity significantly decreased after 55 years black pepper continuous cropping. Fierer et al. [48] found that the soil microbial diversity had been almost completely eradicated with decades of intensely agricultural practices in tallgrass prairie soils of the United States. Similarly, continuous cultivation of cucumber and potato caused reductions in soil richness and diversity indices of soil bacterial communities [14,49]. Soil microbial abundance and diversity have an important role in soil quality, functions, and soil ecosystems sustainability [50]. Hence, the loss of soil microbial abundance and diversity might be contributed to the crop poor growth in continuous cropping systems.

Bray-Curtis dissimilarity hierarchical cluster analysis revealed obvious variations in bacterial community structure across the 3 time-scale replanted black pepper orchards (Fig 3). This result was consistent with the findings of Li et al. [19] that soil microbial community compositions and structures were significantly different among three peanut fields with different monoculture histories using 454 pyrosequencing analysis. Thus, we speculated that soil microbial community could be affected greatly by long-term continuous monocropping. Through the RDA analysis, remarkable variations were found in microbial structure in the 3 time-scale replanted black pepper orchards, probably due to the significant differentiation of soil chemical properties, as soil variables play very important roles in microbial community structure [51,52].

In conclusion, continuous cropping in black pepper cultivation led to significant declines in soil pH, organic matter, soil enzymatic activities, and resulted a decrease in soil bacterial abundance. The 454 pyrosequencing analysis of 16S rRNA genes of bacterial communities from the 3 time-scale replanted black pepper orchards soils revealed that the soil bacterial community membership, diversity, and structure were significantly affected by the black pepper continuous cropping system. All these changes might finally result in black pepper poor growth in the continuous cropping system. Thus, exploring sustainable agricultural measures to improve soil pH, organic matter, soil enzymatic activities, and soil microbiotas are extremely important for the black pepper production in tropical China and will be the focus of our future research.

## Supporting Information

S1 Table Black pepper yields in the 3 time-scale fields. (DOCX)

S2 Table Detail information of the barcode sequence for each sample. (DOCX)

S3 Table Spearman’s rank correlation coefficients (r) between the abundant bacterial phyla and soil properties. (DOCX)
