# Supplementary material for: The Effect of Long-Term Continuous Cropping of Black Pepper on Soil Bacterial Communities as Determined by 454 Pyrosequencing
Source: PLoS One. 2015 Aug 28;10(8):e0136946. doi: 10.1371/journal.pone.0136946 (PMC4552827; doi:10.1371/journal.pone.0136946)
Supplement: S1 Table — (DOCX) [file pone.0136946.s001.docx]

**S1 Table. Black pepper yields in 3 time-scale fields.**

| Black pepper orchards | Average dry weight of black pepper in the past three years^§^ (kg/ha) |
| --- | --- |
| 10y | 321.33±25.54 a |
| 21y | 263.00±19.47 b |
| 55y | 244.67±18.77 b |

“10y”, “21y”, and “55y” stand for 3 black pepper orchards with 10, 21, and 55 years’ succession cropping history, [respectively](app:ds:respectively).

^§^indicate the years of 2010, 2011, and 2012.

Values are means ± standard deviation (n=3).

Means followed by the same letter for a given factor are not significantly different (*P* < 0.05; Turkey’s HSD test).
