# Supplementary material for: The Effect of Long-Term Continuous Cropping of Black Pepper on Soil Bacterial Communities as Determined by 454 Pyrosequencing
Source: PLoS One. 2015 Aug 28;10(8):e0136946. doi: 10.1371/journal.pone.0136946 (PMC4552827; doi:10.1371/journal.pone.0136946)
Supplement: S2 Table — (DOCX) [file pone.0136946.s002.docx]

**S2 Table. Detail information of the barcode sequence for each sample.**

| Sample | Barcode sequence |
| --- | --- |
| 10y-1 | ATCTATA |
| 10y-2 | ATGCCAC |
| 10y-3 | ATGCGTC |
| 21y-1 | CGCTGTC |
| 21y-2 | CGTCCGT |
| 21y-3 | CTCAGCT |
| 55y-1 | GACGTAC |
| 55y-2 | GCATCAC |
| 55y-3 | GCCTAGA |

“10y”, “21y”, and “55y” stand for 3 black pepper orchards with 10, 21, and 55 years’ succession cropping history, [respectively](app:ds:respectively).
