# Supplementary material for: The Effect of Long-Term Continuous Cropping of Black Pepper on Soil Bacterial Communities as Determined by 454 Pyrosequencing
Source: PLoS One. 2015 Aug 28;10(8):e0136946. doi: 10.1371/journal.pone.0136946 (PMC4552827; doi:10.1371/journal.pone.0136946)
Supplement: S3 Table — (DOCX) [file pone.0136946.s003.docx]

**S3 Table.** **Spearman’s rank correlation coefficients (r) between** **the abundant bacterial phyla and soil properties.**

|  | pH | OM | Available N | Available P | Available K |
| --- | --- | --- | --- | --- | --- |
| *Acidobacteria* | -0.517 | -0.6 | 0.617 | 0.517 | -0.509 |
| *Proteobacteria* | 0.683* | 0.45 | -0.467 | -0.4 | 0.509 |
| *Bacteroidetes* | 0.483 | 0.933** | -0.733* | -0.933** | 0.949** |
| *Actinobacteria* | 0.033 | -0.117 | -0.333 | -0.017 | -0.186 |
| *Planctomycetes* | -0.817** | -0.433 | 0.483 | 0.633 | -0.559 |
| *Firmicutes* | 0.583 | 0.950** | -0.783* | -0.850** | 0.881** |
| *Chloroflexi* | -0.767* | -0.567 | 0.467 | 0.5 | -0.525 |
| *Gemmatimonadetes* | -0.333 | -0.4 | 0.433 | 0.383 | -0.492 |
| *Nitrospira* | -0.283 | -0.283 | 0.333 | 0.2 | -0.254 |

* indicate significant correlations (*P* < 0.05), ** indicate significant correlations (*P* < 0.01).
